# Comparative analysis of alignment-free genome clustering and whole genome alignment-based phylogenomic relationship of coronaviruses

**DOI:** 10.1371/journal.pone.0264640

**Published:** 2022-03-08

**Authors:** Anastasiya D. Kirichenko, Anastasiya A. Poroshina, Dmitry Yu. Sherbakov, Michael G. Sadovsky, Konstantin V. Krutovsky

**Affiliations:** 1 Department of Genomics and Bioinformatics, Institute of Fundamental Biology and Biotechnology, Siberian Federal University, Krasnoyarsk, Russian Federation; 2 Laboratory of Molecular Systematics, Limnological Institute, Siberian Branch of Russian Academy of Sciences, Irkutsk, Russian Federation; 3 Faculty of Biology and Soil Studies, Irkutsk State University, Irkutsk, Russian Federation; 4 Novosibirsk State University, Faculty of Natural Sciences, Novosibirsk, Russian Federation; 5 Institute of Computational Modelling, Siberian Branch of Russian Academy of Sciences, Krasnoyarsk, Russian Federation; 6 V.F. Voino-Yasenetsky Krasnoyarsk State Medical University, Krasnoyarsk, Russian Federation; 7 Federal Research and Clinical Center, Federal Medical-Biological Agency, Krasnoyarsk, Russian Federation; 8 Department of Forest Genetics and Forest Tree Breeding, Georg-August University of Göttingen, Göttingen, Germany; 9 Center for Integrated Breeding Research, Georg-August University of Göttingen, Göttingen, Germany; 10 Laboratory of Forest Genomics, Genome Research and Education Center, Institute of Fundamental Biology and Biotechnology, Siberian Federal University, Krasnoyarsk, Russian Federation; 11 Laboratory of Population Genetics, N.I. Vavilov Institute of General Genetics, Russian Academy of Sciences, Moscow, Russian Federation; 12 Scientific and methodological center, G. F. Morozov Voronezh State University of Forestry and Technologies, Voronezh, Russian Federation; Universite du Quebec a Montreal, CANADA

## Abstract

The SARS-CoV-2 is the third coronavirus in addition to SARS-CoV and MERS-CoV that causes severe respiratory syndrome in humans. All of them likely crossed the interspecific barrier between animals and humans and are of zoonotic origin, respectively. The origin and evolution of viruses and their phylogenetic relationships are of great importance for study of their pathogenicity and development of antiviral drugs and vaccines. The main objective of the presented study was to compare two methods for identifying relationships between coronavirus genomes: phylogenetic one based on the whole genome alignment followed by molecular phylogenetic tree inference and alignment-free clustering of triplet frequencies, respectively, using 69 coronavirus genomes selected from two public databases. Both approaches resulted in well-resolved robust classifications. In general, the clusters identified by the first approach were in good agreement with the classes identified by the second using *K*-means and the elastic map method, but not always, which still needs to be explained. Both approaches demonstrated also a significant divergence of genomes on a taxonomic level, but there was less correspondence between genomes regarding the types of diseases they caused, which may be due to the individual characteristics of the host. This research showed that alignment-free methods are efficient in combination with alignment-based methods. They have a significant advantage in computational complexity and provide valuable additional alternative information on the genomes relationships.

## Introduction

Coronaviruses are a large family of single-stranded RNA viruses that infect many animal species, including humans, causing respiratory, gastrointestinal, hepatic, and neurological diseases [1]. This family is the largest known family of RNA viruses and is divided into four genera: alpha-, beta-, gamma-, and deltacoronaviruses, respectively [2].

Alpha- and betacoronaviruses infect only mammals, usually causing respiratory diseases in humans and gastroenteritis in animals. Gamma and delta coronaviruses infect birds, but some of them can also infect mammals [3]. Alpha- and betacoronaviruses can cause severe diseases in livestock. These viruses include swine vector-borne gastroenteritis virus [4], swine intestinal diarrhea virus (PEDV) [5], swine acute diarrhea syndrome coronavirus (SADS-CoV) [6], and some others.

To date, seven human coronaviruses (HCoVs) have been identified: two alphacoronaviruses HCoVs-NL63 and HCoVs-229E and five betacoronaviruses HCoV-OC43, HCoV-HKU1, SARS-CoV, MERS-CoV and SARS-CoV-2. The latter three coronaviruses can cause severe respiratory syndrome [7, 8], while the former four usually cause only mild upper respiratory tract diseases. At the same time, some of them can cause severe infections in infants, young children, and the elderly. Modern ideas about the origin of highly pathogenic strains of human coronaviruses suggest that they all had animals as the primary hosts: supposedly bats for SARS-CoV, MERS-CoV, HCoV-NL63 and HCoV-229E, and rodents for HCOV-OC43 and -HKU1 [9–11]. A new supposedly canine-feline recombinant and potentially dangerous for humans alphacoronavirus CCoV-HuPn-2018 has been discovered recently [12], but its genome was not included in our study presented here.

The SARS-CoV-2 pandemic has dramatically increased interest in this entire virus family. The genome of SARS-CoV-2 is 96% identical to that of the RaTG13 coronavirus isolated from bat droppings, which supports a hypothesis that bats are the most likely primary host of SARS-CoV-2 [13, 14]. However, various assumptions have been also made about potential intermediate host of this virus including snakes [15], lizards [16], minks [8] and pangolins [17, 18], but an intermediate host has not been found yet, and there may be several hosts [19]. Meanwhile, the identification of the natural carrier of the virus is very important to control its spread.

The genomes of coronaviruses are relatively short and similar enough to succeed a reliable whole genome alignment and build a phylogeny based on it. However, this method is very time-consuming, which makes important to explore alternative methods. In addition, such alternative methods can detect connections between genomes that are not detected by alignment. For example, a comparison of the complete mitochondrial genomes of animals by triplet frequencies not only revealed a very strong relationship between the classes allocated in the frequency space by the unsupervised classification (using the method of dynamic nuclei) and traditional animal taxonomy [20], but also between the function of the encoded gene and taxonomy [21–23].

The main objective of the presented study was to compare two methods for identifying relationships between coronavirus genomes: phylogenetic one based on the whole genome alignment and alignment-free clustering of triplet frequencies, respectively. It should be emphasized here that we searched not as much for agreement between these two methods, as rather for meaningful differences in the phylogenetic relationships and signals that they reveal.

## Materials and methods

### Coronavirus genome sequences

In total, 69 genome sequences of coronaviruses (family *Coronaviridae*, subfamily *Orthocoronavirinae*) representing all four genera were selected in this study: 19 alphacoronaviruses including two (NL63 and 229E) affecting humans and eight affecting bats, 24 betacoronaviruses including five (OC43, HKU1, SARS-CoV, MERS-CoV and SARS-CoV-2) affecting humans, two affecting pangolins, and three affecting bats, 6 gammacoronaviruses, 10 deltacoronaviruses and 10 unclassified. Most of the sequences were taken from NCBI GenBank [24]. Three SARS-CoV-2 genome sequences of coronaviruses that emerged in June 2020 in Beijing, China and RaTG13 virus sequences were taken from GISAID database [25].

The set of sequences used in the study was arranged in such a way that the main clades of coronaviruses were represented uniformly, and sequences were more than 95% complete. They are listed in S1 Table. The data for the analysis were chosen among high quality sequences (with the amount of ambiguities less than 5%) so that 1) all major coronavirus taxa represented, 2) branching points on the tree were approximately uniformly distributed on the phylogeny, 3) number of sequences must be small enough not to impede phylogenetic inferences with homoplasies and not to bias the model of molecular evolution due to too many close sister sequences, and still big enough to obtain unbiased estimates of *k*-mer frequencies.

### Phylogenetic analysis based on whole genome alignment

Sequence alignment was performed using MAFFT (version 7 [26]), and the universal iterative refinement algorithm L-INS-i suitable for sequences saturated with extended indels. The search for the optimal model of molecular evolution and phylogenetic constructions were carried out using the IQ-TREE program version 1.6.12 [27] and a version of the GTR (General Time Reversible) model of molecular evolution, in which some sites are invariant, and the rest follow the gamma distribution (GTR+I+G).

The reliability of the tree topology was evaluated using the IQ-TREE method of approximate estimation of the LRT—aLRT (approximate likelihood-ratio test) based on the idea of a conventional LRT with a null hypothesis assumption that the assumed branch has a zero length. This test is fast because the value of the logarithm of likelihood is calculated by optimizing only for the branch of interest and four adjacent branches, while the other parameters are fixed to their optimal values corresponding to the best ML tree.

The analysis and visualization of the results were carried out using original programs in the programming languages R [28] and Python [29]. As a measure of the evolutionary distance between genomes, two types of patristic indicators measured along the branches of the tree were used: the sum of the branch lengths and the number of nodes between the leaves (terminal branches) of the tree, respectively. To calculate the matrix of pairwise patristic distances of both types, the ETE-3 package for Python was used [30].

**Fig 1:**
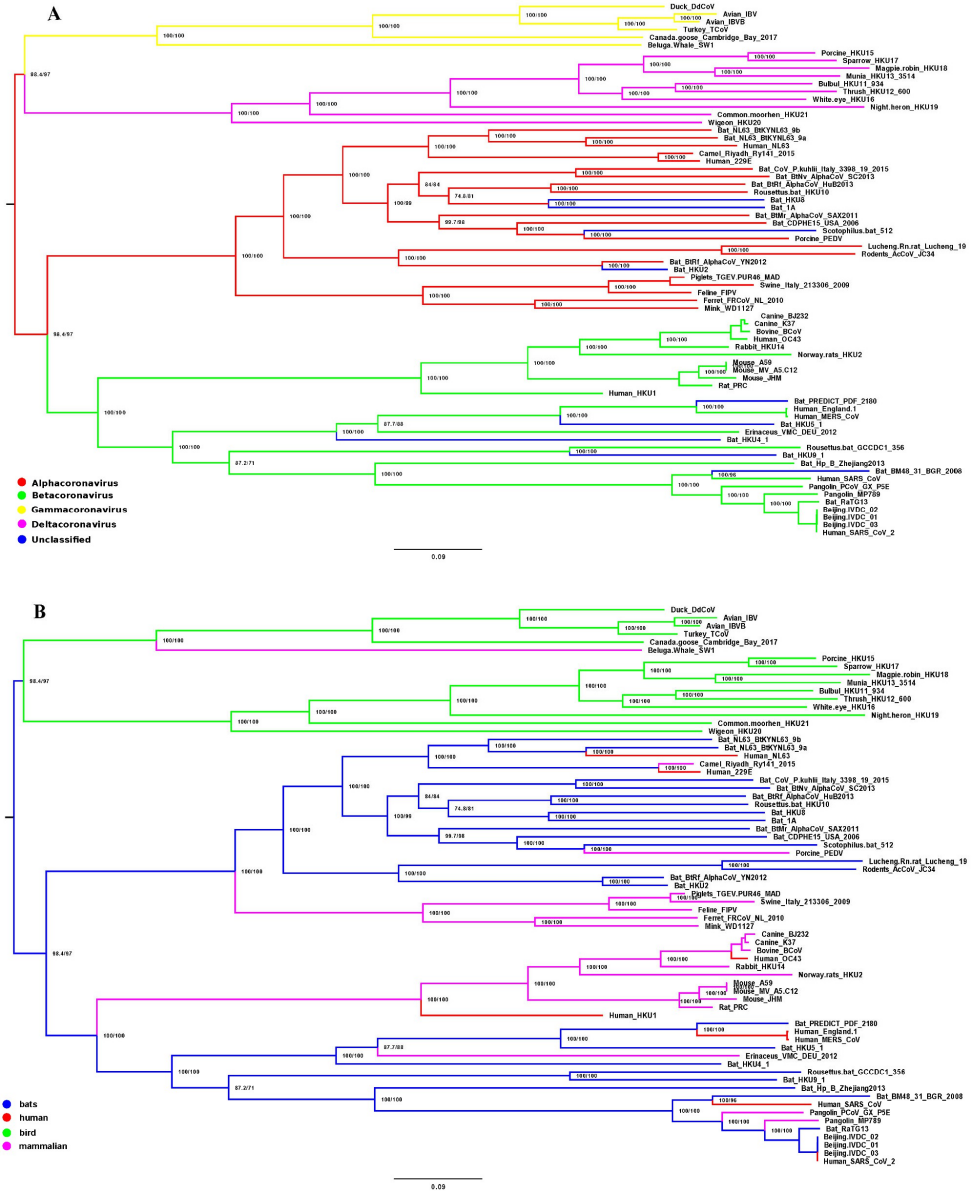
Phylogenetic tree based on the alignment of 69 whole genome coronavirus sequences. Different colors highlight taxonomic affiliation of the coronaviruses (A) and the type of the disease caused by them (B).

The phylogenies inferred were visualized with program FigTree version 1.4.4 [31] and Splitstree version 5.0.20 [32]. On the resulting phylogenetic tree, the identities of the genomes were highlighted by different colors according to their taxonomic affiliation to one of four genera of coronaviruses (Fig 1A) and to the type the disease that they caused (Fig 1B). The taxonomic affiliation and type of disease were determined according to the annotation in the NCBI GenBank taking into account also data from available publications and primary descriptions (S1 Table).

Tests involving tree topology randomization, ancestral state deduction and obtaining metrics of correspondence between tree topology and the most probable evolutionary history of a trait, were performed using Mesquite v. 3.61 [33]. As a measure of correspondence between tree and a trait the most parsimonious number of steps required to explain that changes of trait states on the tree was used. This metric may be treated as a number of homoplasies on the tree with respect to a trait. Higher minimal number of steps means more homoplasies and thus less correspondence between the trait and phylogeny. In other words, the trait in this case changes its state faster and easier than the characters used for the tree inference.

We have examined two traits regarded as the multistate qualitative ones: taxonomic identity of coronaviruses (expected to follow closely the molecular phylogeny) and type of disease that they cause. The values of these traits are summarized in S1 Table. Permutation test according to [34] was used to determine whether phylogenetic signals are present for two selected traits. It involved obtaining a set of fully resolved tree topologies sampled from the treespace for the same number of OTUs. Due to the vast volume and complex structure of the set [35] it has been sampled from a reasonably wide set of topologies by repeating 100 random branch-and-bound iterations starting from the initial topology to obtain 105 replicates. For each replicate, the minimal number of steps (trait’s state changes) was calculated, and the distribution of these numbers was built. This distribution was compared to the minimal number of steps on the molecular phylogeny inferred from the genomic sequences.

The initial minimum number of steps calculated for the type of disease caused by coronavirus was 20 (the number of possible states = 6), and 12 (the number of possible states = 5) for taxonomic identity.

### Generating triplet frequency dictionaries

Each genome is a sequence from four letter alphabet ℵ = {A, C, G, T}; let *N* be the length of a sequence. A triplet frequency dictionary is the list of all possible triplets *ω* = *v*_1_*v*_2_*v*_3_, from *ω* = *AAA* to *ω* = *TTT*. The number (integer) of each triplet in the sequence was calculated by moving a reading frame with a three nucleotide window and a three nucleotide step from the first till the last nucleotide in a genome sequence. In total, there are 64 possible triplets. The reading frame may move along a sequence with different step, but we used the step equaled three nucleotides, so that there was no overlap for any two triplets. However, no gap between the neighboring triplets took place, either. Changing the number of copies *n*_*ω*_ for The frequency *f*_*ω*_ of the triples (frequency dictionary) was calculated as $\;f_{\omega }=\frac{n_{\omega }}{N}$, where *N* was a total number of nucleotides in a genome. Thus, each genome can be presented as a point in a 64-dimensional (64D) space [36] (more rigorous definitions see in [37, 38]).

There are some other sizes for *k*-mer that could be explored, but it is beyond the scope of this study. We selected *k* = 3 for two reasons: a triplet is highly biologically distinguished length of strings, and longer strings bring the curse of dimensionality problem. It is important to emphasize here that alignment-free approach involves other properties of a genome than just its linear nucleotide sequence. Some other properties of viral genome (for example, packing or unfolding affecting the genome nucleotide structure) may be better seen with an alignment-free method.

Two genomes are regarded as much close to each other as the Euclidean distance between them in 64D space is short. This distance may also be used for a comparison to other measures of distance such as a most-likely mutational distance between the two genomes or some sort of patristic distances obtained as described above. Therefore, the results of the two methods of the assessment of a set of 69 genomic sequences were compared via comparison of two arrays of pairwise distances—patristic and Euclidean distances. These comparisons were visualized graphically as scatter plots built using custom scripts in R and/or Python.

### Clustering of genomes by *K*-means using triplet frequency dictionaries

*K*-means is the simplest and most widely used iterative classification (clustering) algorithm that divides a set of data into *k* classes located at particular distances from each other [39]. The algorithm of the method basically divides the set of points represented in an *n*-dimensional space (in our case, 63-dimensional, since the sum of all frequencies makes one) into *k* classes, with each point initially falling into a randomly determined class. Next, for each class the arithmetic mean is calculated, and the points are checked for the proximity to a class using the Euclidean distance. Then, if a point initially belonging to class *j* becomes close to the kern (the arithmetic mean) of the class *m*, then the point changes the class attribution. Such rearrangement iterates till the stop. As soon as the new distribution is obtained, the kerns (arithmetic means) are recalculated, and the procedure runs again. The entire algorithm is repeated until the transition of points between classes stops.

The freely distributed ViDaExpert software was used to implement *K*-means [40]. In this study, we tested different number of clusters by iterating over the reasonable values of *K* from 2 to 11, but we did not aim to find the best possible optimal number of clusters. The key question of the study was the relation between the class compositions observed for various *K*.

### Elastic map clustering

The elastic map was also used for nonlinear dimensionality reduction and visualization of multidimensional data in this study [41–43]. The method essentially consists of the following three steps: 1) the multidimensional data space is generated (in our case, it was 63-dimensional because the CGA triplet was excluded from the analysis due to its smallest contribution to the distinctness of genomes as suggested by its standard deviation for the entire set of genomes); 2) an elastic plane is added, stretched and bent so that it becomes the closest possible to all points while remaining minimally curved and stretched (in other words, it is deformed in such a way as to approximate the available data points and at the same time to be not too curved and stretched); 3) orthogonal projections of the data points are defined and projected onto the two-dimensional plane, and displayed on it as on a map.

More rigorously, the method proceeds as follows. At the first step, the first and the second principal components are calculated, and a plane is built on them as on the axes. Then, all the data points are projected onto this plane, and the minimum square containing all the points is determined. A square is divided by a certain number of smaller squares (16 and 25 are used in our study). In the second step, each data point is connected by a mathematical spring to the lattice node closest to the projection. Then, the rigid plane (more precisely, the part of it that corresponds to the larger square) is replaced by an elastic membrane, with the elasticity being uniform. In the third step, the entire system is released, and the springs are reduced (or stretched being affected by the membrane and neighboring springs), so that the membrane is deformed. In this case, the deformation of the membrane and its final state are determined by the minimum of the total deformation energy. In the fourth step, the position of each point on the deformed map is redefined, and a new orthogonal projection is found (the point on the deformed map that is closest to the original). Finally, all the mathematical springs are removed, and the deformed membrane returns to its original flat state; the point images also change their position on the elastic map. This transformation is called a transition to the internal coordinates.

It should also be noted that the elasticity of the map is selected "manually". The more elastic the map, the smoother the model it represents (for large values of the elasticity coefficient, the map nodes are practically in the same plane, and this is the plane of the principal components).

For the studied genomes, the distribution of points corresponding to frequency dictionaries in the frequency space was constructed. The definition of the cluster on the elastic map, represented in internal coordinates, was calculated from the local density. To do this, each point on the elastic map (recall the image of the original data point) was provided with a bell-shaped function; it is clear that the choice of functions of this type is very wide, but we used the Gaussian function. Then, the values of all the functions for each point were summed over all the points, and the value of the total function determined the local density. Displaying this function, we used a scheme with 15 levels of the value of the local density function; the cluster was considered to be an area with a local density exceeding the 9th level from below. This is a free parameter and is chosen expertly, similar to the penalties for matching and mismatch in alignment. The freely distributed ViDaExpert software was also used to implement this method [40].

### Comparison of elasic distance based clustering of genomes to phylogenies

Pair-wise patristic distances between all genomes involved into analysis were calculated from phylogeny described above using Python library ete3 [30]. Two different metrics for the patristic distance were used, further designated as “Patristic 1” and “Patristic 2”, based on the sum of lengths of all vertices connecting the tips (“Patristic 1”) and the number of internal nodes on the way from one tip to the other (“Patristic 2”), respectively. Euclidean distance between the points on the “elastic map” were used for comparison with patristic distance. Data in the 63-dimensional Euclidean space were twice non-linearly transformed and projected onto a flat map, while maintaining the real relationship between genomes. We extracted the 2D coordinates of the projections of these points on the map for comparison with the patristic distances.

Custom scripts in R language were used for plotting the pairwise differences as well as for calculation of correlations between the distances. Kendall τ was used in order to minimize possible autocorrelation effects.

Two characters were used to group the sequences: the taxonomic identity of the viruses was chosen as the presumably most phylogenetically informative one, while the kind of disease caused, which was the second trait, was regarded as the most practically important.

## Results

### Phylogenetic inferences from the aligned sequences

A fully resolved phylogenetic tree based on the alignment of 69 whole genome coronavirus sequences was inferred, all internal nodes of which had high support (Fig 1). It should be noted that the internal nodes were approximately evenly distributed along the length of the tree. In general, we can conclude that the topology of this tree is stable enough to compare it with the results of other methods for assessing the similarity/difference of viral genomes, as well as to investigate the evolution of their different traits.

In this study we have analyzed two traits: the taxonomic identity of coronaviruses and type of disease that they cause. The former is expected to follow tree topology closely. This correspondence may only occasionally be spoiled by sequences of misclassified viruses or by low-quality sequences. The natural hosts of the viruses were inferred from the virus description, but should be used conditionally with some reservation.

For each trait the following two hypotheses were tested: 1) the first one assumed that the change in the state of the trait occurs very easily, and the number of homoplasies on the tree is random, 2) the second one assumed that the trait was conservative, and the number of state changes of this trait tends to become as low as possible and thus carry strong phylogenetic signal.

The results of mapping taxonomic and disease characters of coronaviruses on their phylogeny are presented on Fig 1, where the taxonomic identity of the coronaviruses (Fig 1A) and the type of the disease caused by them (Fig 1B) are highlighted by different colors. It turned out that the taxonomy corresponded very well to the resulting tree (most parsimonious number of steps = 27, while median = 33). The amount of phylogenetic signal regarding the type of the disease was somewhat less, although its correspondence to the phylogeny was still strongly present (minimum number of steps = 29, median = 35). However, it should be noted that the type of disease is a highly variable trait and depends strongly on the individual characteristics of the host. Still this analysis points at high flexibility of coronaviruses with respect to disease they cause and suggests poor predictive power of phylogeny when trying to predict the form of disease caused by new variants of coronaviruses.

### Analysis of the triplet frequencies

#### Classification of genomes by *K*-means

**Fig 2:**
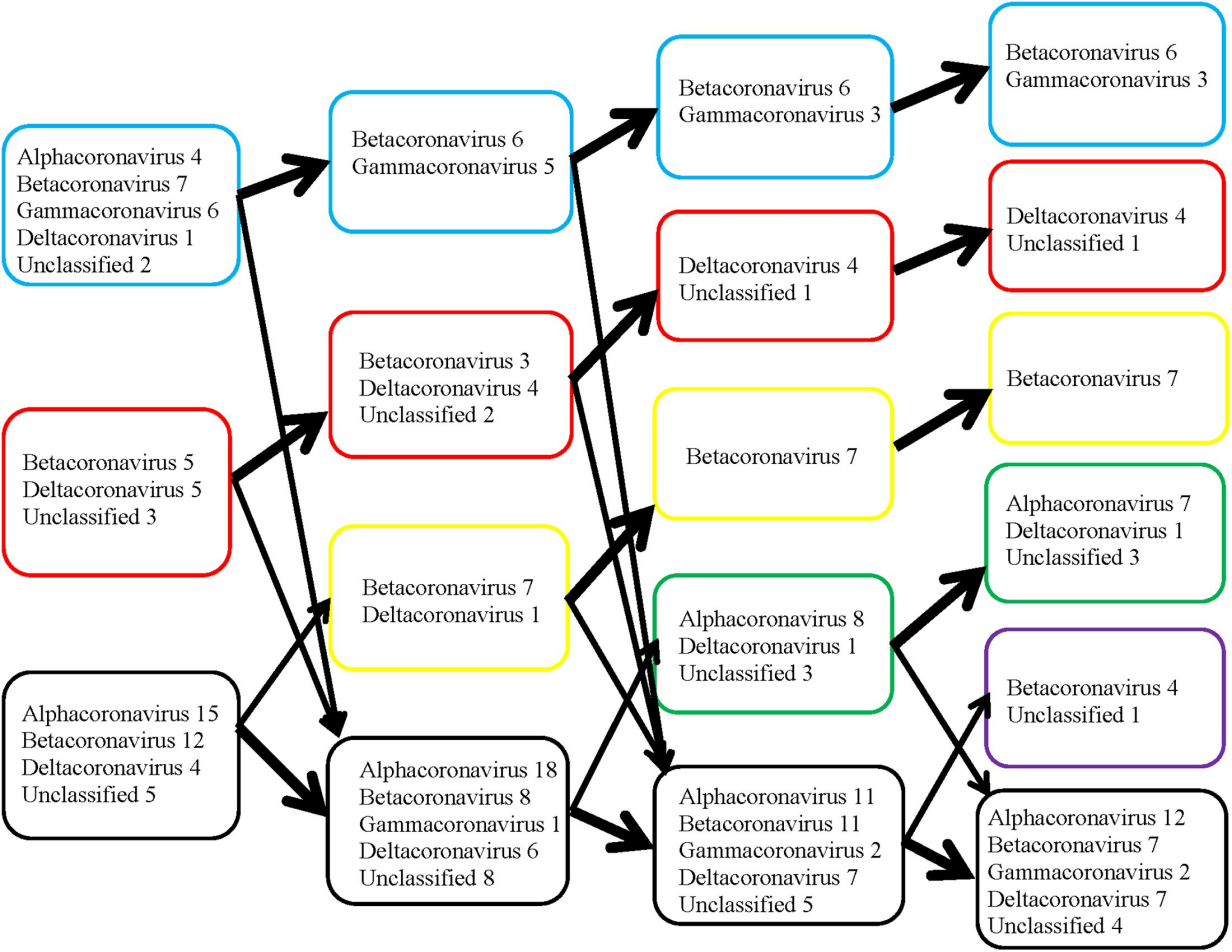
Results of step-by-step classification of 69 coronavirus genomes belonging to different genera by *K*-means (from 2 to 5 classes). Boxes in a blue frame represents the 1st class, red—2^nd^ class, yellow—3^rd^, green—4^th^, violet—5^th^, black—unclassified volatile genomes, the position of which was unstable between classes.

**Fig 3:**
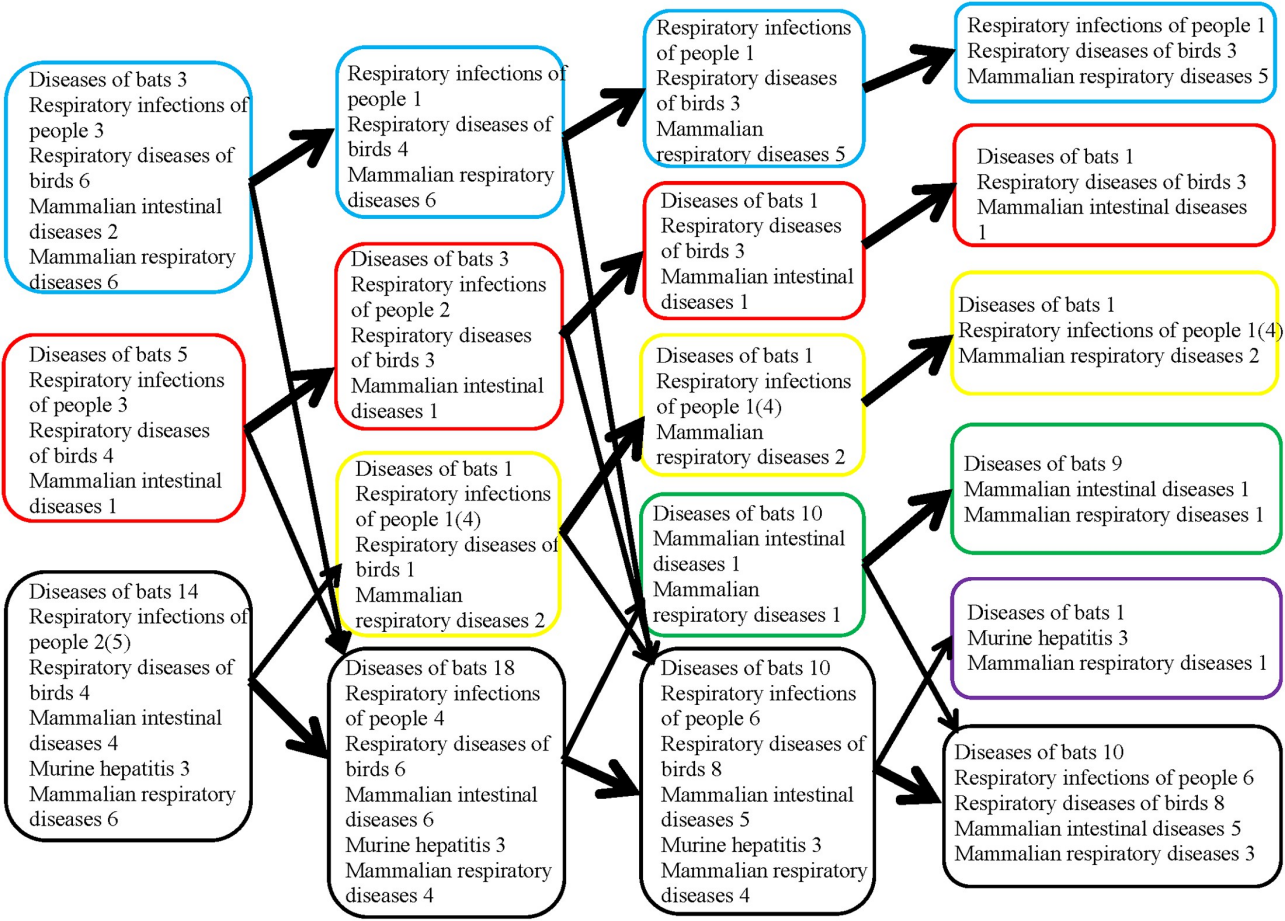
Results of the same step-by-step classification of 69 coronavirus genomes by *K*-means (from 2 to 5 classes) as in Fig 2 of the same viruses but named according to the type of disease that they cause. Boxes in a blue frame represents the 1^st^ class, red—2^nd^ class, yellow—3^rd^, green—4^th^, violet—5^th^, black—unclassified volatile genomes, the position of which was unstable between classes.

A separate data file was generated for the genome sequences according to the data format for ViDaExpert software. The step-by-step classification was carried out by *K*-means from 2 to 5 classes (Figs 2 and 3).

When the genomes were divided into two classes, two stable groups of genomes were formed highlighted by blue (1st class) and red (2nd class) frames in Fig 2, respectively. In 98 out of 100 cases the 1st class was formed by the following genomes: all six genomes of gammacoronaviruses presented in our sample, four alphacoronaviruses (human NL63, bat BtRf_AlphaCoV_YN2012, ferret FRCoV_NL_2010, and mink WD1127), seven betacoronaviruses (Erinaceus VMC_DEU_2012, rabbit HKU14, bovine BCoV, canine BJ232 and K37, and human HKU1 and OC43), one deltacoronavirus (common moorhen HKU21), and two unclassified bat coronaviruses (HKU4 and HKU2).

When the genomes were divided into three classes, in 86 out of 100 cases only six out of seven betacoronaviruses (human HKU1 was separated) and five out of six gammacoronaviruses (duck DdCoV was separated) remained in the 1st class.

With a further increase in the number of classes, the 1st class included the same six betacoronaviruses and only three avian gammacoronaviruses (IBV, IBVB, and TCoV) in 89 out of 100 cases when dividing the genomes into four classes and in 79 out of 100 cases when dividing into five classes.

The second stable group (2nd class) in 96 cases out of 100 included five betacoronaviruses (three human SARS_CoV, MERS_CoV, and England_1 and two bat Hp_B_Zhejiang2013 and Rousettus.bat_GCCDC1_356), five deltacoronaviruses (four avian—sparrow HKU17, munia HKU13, white-eye HKU16, and magpie-robin HKU18, and one porcine—HKU15), and three unclassified bat viruses (BM48_31_BGR_2008, HKU5_1, and PREDICT_PDF_2180).

When divided into three classes, this group decreased. Human SARS_CoV, Rousettus.bat_GCCDC1_356, white-eye HKU16 and bat BM48_31_BGR_2008 moved to the group of “nomadic” (unclassified volatile) genomes. The stability of this 2nd class has also decreased to 78%. However, when the genomes were divided into four and five classes, the stability of this 2nd class has increased to 95% and 87%, respectively, and consisted of the same four deltacoronaviruses (three avian—sparrow HKU17, munia HKU13 and magpie-robin HKU18, and a porcine—HKU15) and one unclassified bat coronavirus HKU5_1, which was included in the clade of betacoronaviruses based on the phylogenetic tree in Fig 1A.

It is worth noting that among the unstably classified genomes, two groups were formed from the very beginning, which may later form their own stable classes.

When dividing the genomes into three classes, a stable group (3rd class) was emerged from the unclassified group consisting of the seven betacoronaviruses of especially high interest—three human SARS-CoV-2 (reference and three Beijing genomes), two pangolin—MP789 and PCoV_GX_P5E and a bat—Yunnan/RaTG13/2013. This 3rd class included also the avian deltacoronavirus thrush HKU12_600, which left this group and moved back to the unclassified group with a subsequent increase in classes, while the 3rd class with seven betacoronaviruses remained unchanged and stable (in 100 out of 100 cases), when further groups were divided into four and five classes.

The 4th class was formed mainly by the genomes of bat coronaviruses: eight alphacoronaviruses including six bat (BtNv_AlphaCoV_SC2013, BtMr_AlphaCoV_SAX2011, CDPHE15_USA_2006, CoV_P.kuhlii_Italy_3398_19_2015, NL63_BtKYNL63_9a, NL63_BtKYNL63_9b) and two rodent (rat Lucheng.Rn.rat_Lucheng_19 and mouse AcCoV_JC34) viruses, and three unclassified bat viruses (HKU8, Scotophilus.bat_512 and HKU2), which according to the phylogenetic tree in Fig 1A were included in the clade of alphacoronaviruses. This group included also a deltacoronavirus of porcine PEDV. The stability of this class was 75%. When the genomes were divided into five classes, this 4th class became more stable (87 out of 100 cases), but bat virus NL63_BtKYNL63_9a left the group.

The last 5th class emerged from a group of unstably classified genomes and included genomes of four rodent betacoronaviruses (three mouse—A59, MV_A5.C12 and JHM, and one rat—PRC) and the unclassified bat genome HKU9_1, which was included in the betacoronavirus clade, according to the phylogenetic tree in Fig 1A. Before they formed the 5th class, these genomes moved together from class to class in 96–97% of cases. Classification of coronaviruses in the same classes as in Fig 2 but named according to the type of disease that they cause is presented in Fig 3.

#### Elastic map clustering

**Fig 4:**
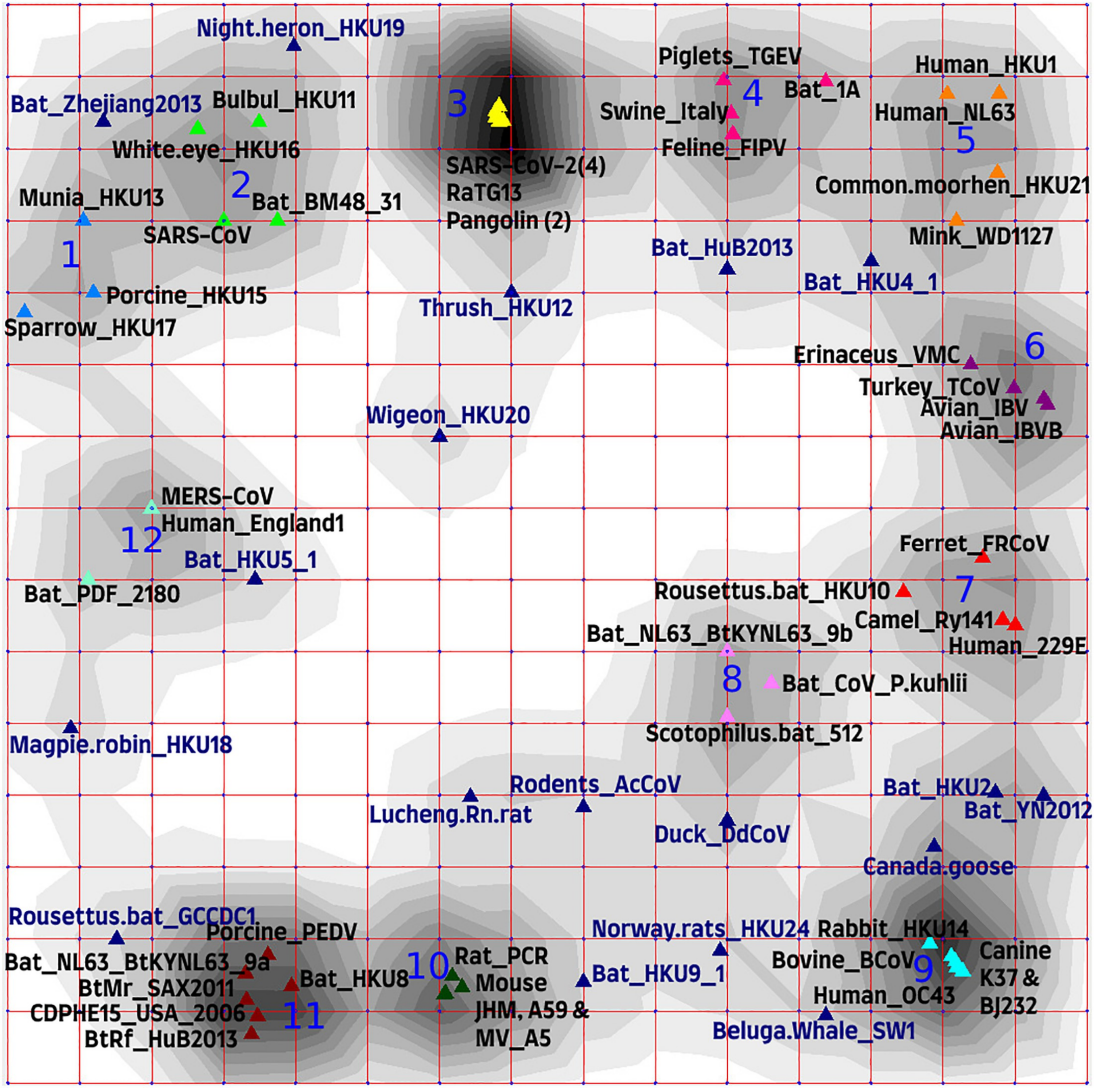
Elastic map of the 16×16 type in internal coordinates reflecting local density by shades of grey contours and demonstrating 69 coronavirus genomes depicted by differently colored triangle dots included in 12 clearly distinguished clusters (1–12); dark blue triangle dots depict unassigned genomes.

Data visualization using the elastic map method gave similar result. Clusters were allocated according to the following two rules: 1) based on the local density levels, and 2) the cluster must include at least 3 points. Fig 4 shows an elastic map of the 16×16 type in internal coordinates with a display of the local density, on which 12 clusters are clearly distinguished.

The genomes in the first cluster (depicted as blue triangle dots) included three deltacoronavirus genomes: two avian—sparrow HKU17 (NC_016992) and finch (munia) HKU13 (NC_011550), and one porcine HKU15 (NC_039208). The radius of this cluster is 0.009. It should be noted that the values of the radius of all clusters were defined in natural coordinates that are the frequencies of triplets.

The second cluster with a radius of 0.01 included four genomes (depicted as light green triangle dots): two avian deltacoronaviruses—bulbul HKU11 (NC_011547) and white-eye HKU16 (NC_016991) birds, and two betacoronaviruses—bat BM48-31/BGR/2008 (NC_014470) and human SARS-CoV (NC_004718). The latter two were also in the same cluster in the phylogenetic tree (Fig 1). They are indeed quite similar and have very similar the receptor-binding domain (RBD), but there were differences in other regions of the S-gene, and the ORF8 was also missing in the bat coronavirus.

The third cluster (yellow triangle dots) is the most interesting, it includes the genomes of betacoronaviruses of pangolin PCoV GX-P5E (MT040336) and MP789 (MT121216), bat RaTG13, and four human SARS-CoV-2 (reference NC_045512 and all three Beijing genomes). The cluster radius was 0.003.

The fourth cluster (pink) had a radius of 0.008 and included four alphacoronavirus genomes: two porcine—transmissible gastroenteritis virus (NC_038861) and swine enteric coronavirus (NC_028806), feline infectious peritonitis virus (NC_002306) and bat coronavirus 1A (NC_010437).

The fifth cluster (orange) had a radius of 0.019 and also included four genomes: two human viruses—betacoronavirus HKU1 (NC_006577) and alphacoronavirus NL63 (NC_005831), bird deltacoronavirus—common moorhen HKU21 (NC_016996) and mink alphacoronavirus WD1127 (NC_023760).

The sixth cluster (purple) also included four genomes: three avian gammacoronaviruses—two chicken infectious bronchitis viruses (NC_048213 and NC_001451) and turkey coronavirus (NC_010800), and a hedgehog (*Erinaceus*) betacoronavirus (NC_039207) and had a radius of 0.007.

The seventh cluster (red) had a radius of 0.007 and also included four alphacoronavirus genomes: camel Ry141 (NC_028752), bat (*Rousettus*) HKU10 (NC_018871), human 229E (NC_002645), and ferret FRCoV (NC_030292).

The eighth cluster (purple) had a radius of 0.007 and included three bat alphacoronavirus genomes: Bat-CoV (NC_046964), NL63-related BtKYNL63-9a (NC_032107) and bat *Scotophilus* coronavirus 512 (NC_009657).

The ninth cluster (blue) had a radius of 0.004 and included five genomes of betacoronaviruses: human OC43 (NC_006213), rabbit HKU14 (NC_017083), bovine (NC_003045) and two canine BJ232 and K37 (KX432213 and JX860640, respectively).

The tenth cluster (dark green) had a radius of 0.003 and included four betacoronavirus genomes, three of which belong to the mouse hepatitis viruses A59, JHM, and MHV-A59 C12 (NC_048217, AC_000192, and NC_001846, respectively), and the rat coronavirus Parker (NC_012936).

The eleventh cluster had a radius of 0.009 and included six alphacoronavirus genomes, one of which was porcine epidemic diarrhea virus PEDV (NC_003436), and the other five belong to bat coronaviruses: CDPHE15/USA/2006 (NC_022103), BtMr-AlphaCoV/SAX2011 (NC_028811), BtNv-AlphaCoV/SC2013 (NC_028833), Miniopterus HKU8 (NC_010438) and NL63 (NC_048216). In is interesting that another NL63-related BtKYNL63-9a sequence was included into the eighth cluster.

The twelfth cluster had a radius of 0.007 and included genomeы of three betacoronaviruses: two human MERS-CoV—England 1 (NC_038294) and HCoV-EMC (NC_019843) and one bat HKU5 (NC_009020).

**Fig 5:**
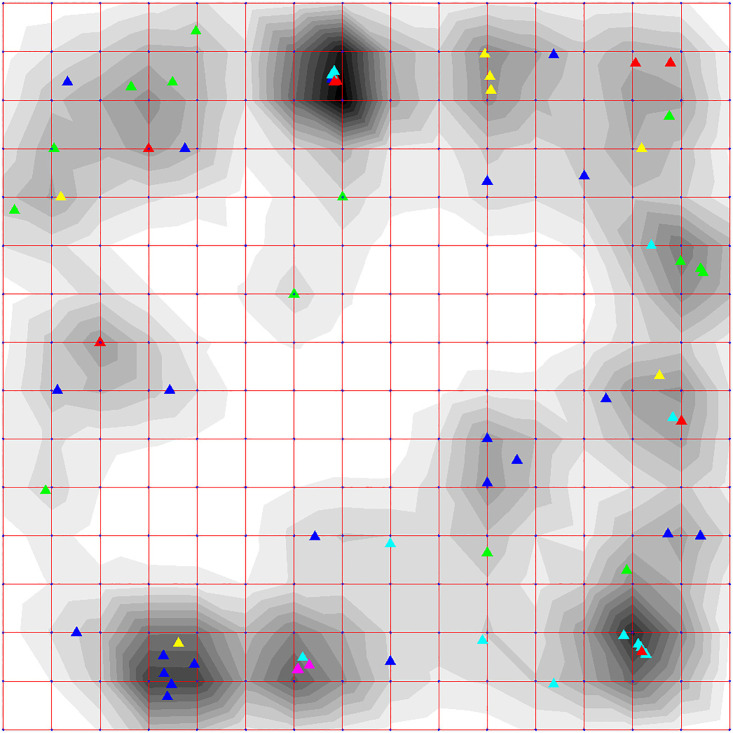
Elastic map of the 16×16 type in internal coordinates reflecting local density by shades of grey contours and demonstrating 69 coronavirus genomes depicted by differently colored triangle dots. Dark blue triangle dots depict viruses that cause disease in bats, red—respiratory disease in humans, light blue—respiratory disease in mammals, yellow—intestinal disease in mammals, pink—hepatitis in mice, and green—respiratory disease in birds.

**Fig 6:**
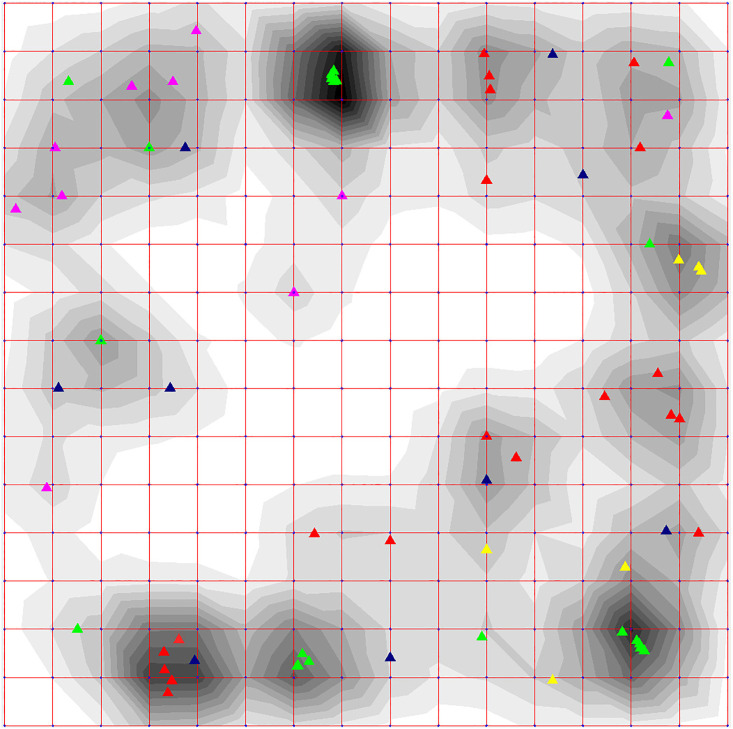
Elastic map of the 16×16 type in internal coordinates reflecting local density by shades of grey contours and demonstrating 69 coronavirus genomes depicted by differently colored triangle dots. Red triangle dots depict alphacoronaviruses, green—betacoronaviruses, yellow—gammacoronaviruses, pink—deltacoronaviruses, and dark blue—unclassified.

In Fig 5, the genomes are highlighted by different colors according to the type of diseases that they cause, respectively, and in Fig 6—according to the taxonomic classification. Table 1 shows the cluster radiuses and the pairwise distances between the cluster centers in Figs 4–6.

**Table 1:** The pairwise distances between the centers of the clusters (*L*) and their radiuses (*R*) on the 16 × 16 elastic map in Figs 4–6.

**L**	**2**	**3**	**4**	**5**	**6**	**7**	**8**	**9**	**10**	**11**	**12**	**R**
**1**	0.020	0.031	0.037	0.058	0.042	0.042	0.039	0.052	0.039	0.037	0.020	0.0091
**2**		**0.013**	**0.019**	0.042	0.026	0.027	0.027	0.037	0.031	0.028	**0.015**	0.0108
**3**			**0.017**	0.039	0.024	0.026	0.028	0.038	0.035	0.033	0.024	0.0032
**4**				0.029	**0.016**	**0.014**	**0.018**	0.025	0.025	0.024	0.024	0.0079
**5**					0.026	0.026	0.031	**0.017**	0.036	0.037	0.045	0.0192
**6**						0.020	0.025	**0.019**	0.022	0.029	0.029	0.0068
**7**							**0.011**	0.024	0.026	**0.019**	0.030	0.0071
**8**								0.028	0.025	**0.011**	0.027	0.0070
**9**									0.024	0.031	0.039	0.0035
**10**										0.023	0.026	0.0031
**11**											0.025	0.0086
**12**												0.0071

The shortest distance values between centers of the clusters are highlighted by the bold font.

### Comparison of the results obtained by phylogenetic analysis based on the whole genome alignment and alignment-free clustering based on triplet frequencies using elastic maps

**Fig 7:**
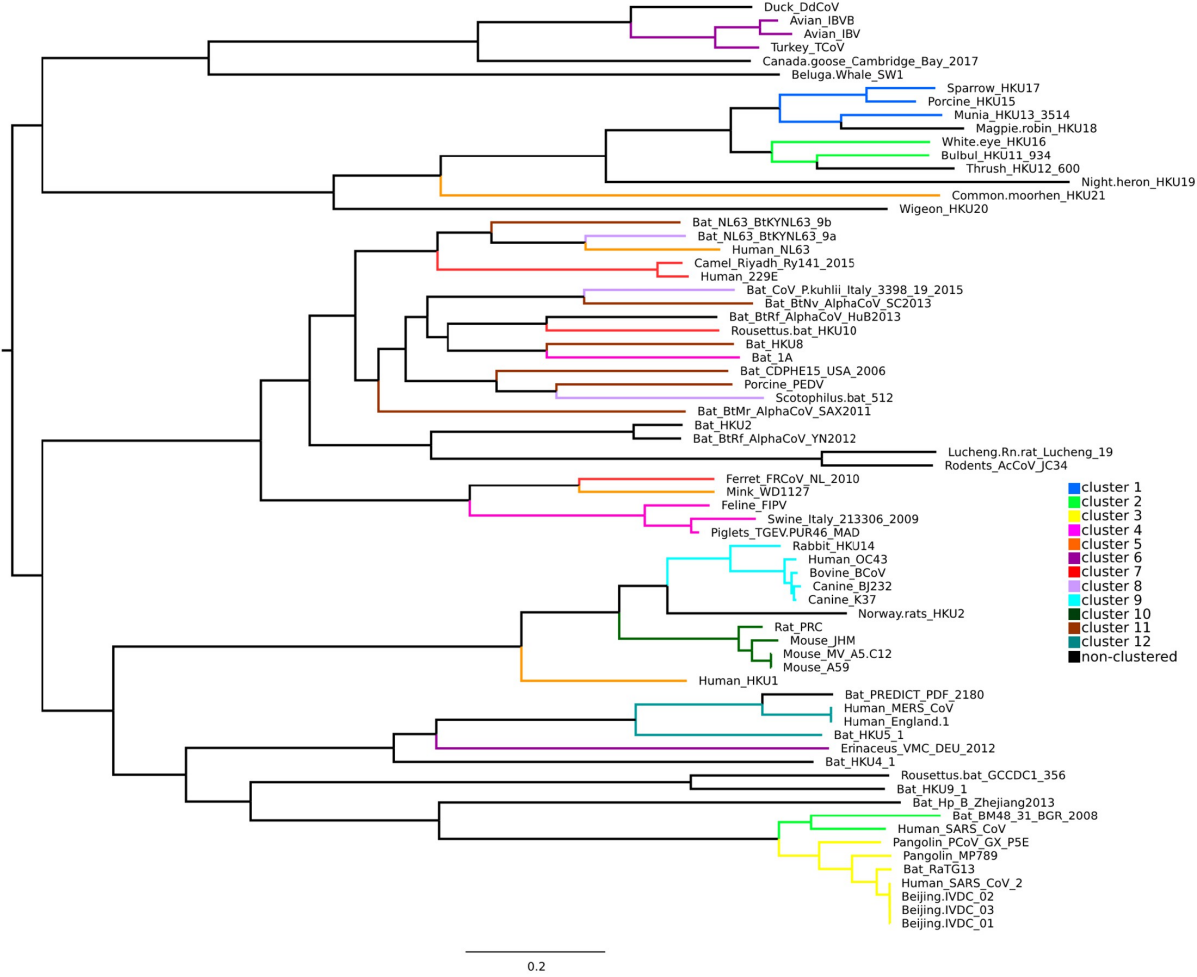
Phylogenetic tree based on whole genome alignment of 69 genomes of coronaviruses (see also Fig 1). Branches of the tree are colored according to the same colors, which were used to color the clusters on the elastic map in Fig 4 and presented in the figure legend also here. Black color depicts genomes that were not included in any cluster on the elastic map.

This study mainly aimed to compare two approaches: alignment-based and alignment-free, respectively, to see if they are in a reasonable agreement in general, and in addition one of them can find phylogenetic relationships, patterns or signals among genomes of coronaviruses that are not found by another. To visualized and compare the obtained patterns, we colored branches of the phylogenetic tree obtained by the traditional method based on the whole genome alignment (Fig 7) using the same colors, which were used to color the clusters on the elastic map in Fig 4.

Cluster 1 on the elastic map was in agreement with the phylogenetic tree confirming that porcine HKU15, sparrow HKU17 and munia HKU13 are very close species, with magpie-robin HKU18 located in a neighboring branch, but not far away. All four genomes were also included in the same class when classified by *K*-means (Fig 2).

The bulbul HKU11 and white-eye HKU16 genomes clustered together on the tree (Fig 7) and corresponded to cluster 2 on the elastic map (coordinates 2C and 2D in Fig 4) and were close to cluster 1 on both the elastic map and the phylogenetic tree. Bat BM48-31 and human SARS-CoV from this cluster 2 on the elastic map were located in another cluster on the tree, but they were adjacent to the viruses from cluster 3, which corresponded to the cluster on the tree that also included four human SARS-CoV-2 genomes together with both pangolin betacoronaviruses and bat RaTG13. All seven genomes in cluster 3 always remained in the same class when classified by *K*-means, while BM48-31 и SARS-CoV from cluster 2 were inconsistent unstably classified genomes.

Piglets TGEV, swine enteric coronavirus, and feline infectious peritonitis virus formed the same cluster 4 on both the tree and the elastic map, but bat coronavirus 1A from this cluster 4 on the elastic map was located far away from these three genomes and joined a completely different group on the tree. Classification by *K*-means agreed with the clustering and phylogenetic relationships of these genomes.

Cluster 5 on the elastic map included genomes that are not related either by the diseases caused by these viruses or by taxonomy, but the radius of this cluster was rather long (0.019), and on the tree, these genomes, as expected, were in completely different clusters. It agreed with classification by *K*-means.

Cluster 6 with a radius of 0.007 included avian coronavirus genomes IBV, IBVB and TCoV, which were also included in the same cluster on the tree, except the hedgehog (Erinaceus) betacoronavirus VMC DEU from the same cluster 6 on the elastic map, which was far from them on the tree and was close to some bat coronaviruses and human MERS-CoV. According to classification by *K*-means, all four genomes were also included in the same one class, which included five more betacoronaviruses in addition to the hedgehog one.

Cluster 7 included the genomes of the camel Ry141 and human 229E alphacoronaviruses, which is quite an interesting and unexpected observation. These two genomes were also in the same cluster on the tree next to the bat coronaviruses. This can also explain their close relationship with Rousettus bat coronavirus HKU10, although the latter was located at a noticeable distance from them on the phylogenetic tree. At the same time, the genome of ferret coronavirus FRCoV from cluster 7 was very far away on the tree from other genomes in this cluster and is grouped with similar viruses that cause intestinal diseases of mammals. When classified by *K*-means, they were also not grouped into one class and represent inconsistent unstably classified genomes.

Cluster 9 included five genomes that also formed a close cluster on the phylogenetic tree. The genomes were consistently included in the same class when classified by *K*-means, which also included avian coronaviruses from cluster 6 and hedgehog betacoronavirus at some distance from them.

Cluster 10 included a group of mouse and rat betacoronavirus genomes and also formed a separate dense cluster on the tree. It is worth emphasizing here that this and the previous clusters were branches of the same large cluster, but on the elastic map they were located at a considerable distance (in internal coordinates) from each other. This group of genomes formed its own class with an increase in the number of classes, which also included the bat HKU9 genome located also in the betacoronavirus branch, but still at some distance from the mouse group of genomes.

Cluster 11 included mostly genomes of bat coronaviruses that were actually all in one large cluster on the tree together with other viruses that infect bats. Particularly noteworthy is the fact that this cluster contained the porcine PEDV genome, which causes mammalian intestinal diseases; it falls into this group both on the elastic map and on the phylogenetic tree and on the classification.

Cluster 12 with radius of 0.007 included only three genomes—two human MERS-CoV viruses and a bat virus HKU5 on the elastic map, and they were also completely included in one cluster on the tree together with another bat virus PREDICT_PDF. Note that when classified by *K*-means, this group of genomes always moved from class to class together.

When exploring clustering on the elastic map one should also take into account not only the distances between the cluster centers, defined in natural coordinates (Table 1), but also the radiuses of the clusters themselves. The mutual relationship of the distances between the centers and the radiuses answers the question of the reliability of the resulting clustering.

The cluster 2 in Fig 4 included two avian deltacoronaviruses, human betacoronavirus SARS-CoV and unclassified bat coronavirus BM48_31, which is very close to SARS-CoV based on the phylogenetic tree, is likely also a betacoronavirus. According to Table 1, this cluster 2 was closest to the cluster 3 in the space of internal coordinates, which included the genomes of SARS-CoV-2, pangolin coronaviruses and RaTG13, and on the phylogenetic tree in Fig 1, next to this branch, there was a pair of SARS-CoV and Bat-BM48_31. In the 63-dimensional space, it was also close to the cluster 12, which included two sequences MERS-CoV and bat PREDICT_PDF_2180, which was also included in the betacoronavirus clade on the phylogenetic tree. At the same time, the center of the cluster 1, formed by three deltacoronaviruses, was equidistant from the centers of the clusters 2 and 12.

Clusters formed by alpha-coronaviruses (4, 7, 8 and 11) had small distances between the centers of the clusters in the 63-dimensional space. The cluster 4 was also quite closely adjacent to the clusters 2 and 3, which may be of interest for further consideration.

From the point of view of the distribution of genomes on the elastic map, the cluster 5 was also very interesting and included not well-connected genomes of human alphacoronavirus NL63, human betacoronavirus HKU1, common moorhen deltacoronavirus HKU21 and mink alphacoronavirus WD1127. This cluster had the largest radius, and the cluster 9 was the closest cluster in the 63-dimensional space (Table 1, Fig 6), which consisted exclusively of betacoronaviruses including human OC43. Interestingly, the cluster 9 was also quite close to the cluster 6, which included three avian gammacoronaviruses and hedgehog betacoronavirus VMC. According to Fig 7, gammacoronaviruses were also present in the vicinity of the cluster 9, which allows us to assume that gammacoronaviruses and betacoronaviruses causing respiratory diseases in mammals (including human OC43) had a similar triplet composition. It is also worth noting that the group of betacoronaviruses in cluster 6 was somewhat distant from betacoronaviruses in the clusters 2, 3 and 12.

The tenth cluster, which consisted of a group of coronaviruses that cause hepatitis in mice and rats, had a rather small radius and was located at a distance from all other clusters.

A detailed 25 × 25 elastic map was also generated, and the number of clusters has decreased to 10. Although, some of the clusters obtained using the soft elastic map did not necessarily merge into one cluster on the detailed map: some of them merged into one, and some broke up into new ones, a similar clustering was observed in general. The discussion of these data seems beyond the main scope of this paper and, therefore, are not presented here.

### Comparison of pairwise Euclidian distances between individual sequences on the elastic map with patristic distances inferred from the phylogenetic tree

To test correspondence in clustering of the coronavirus genomes on the elastic map and on the phylogenetic tree we compared the Euclidian distances between individual genome sequences on the elastic map, with patristic distances inferred from the phylogenetic tree and optimized in the process of phylogenetic analysis using IQ-TREE.

**Fig 8:**
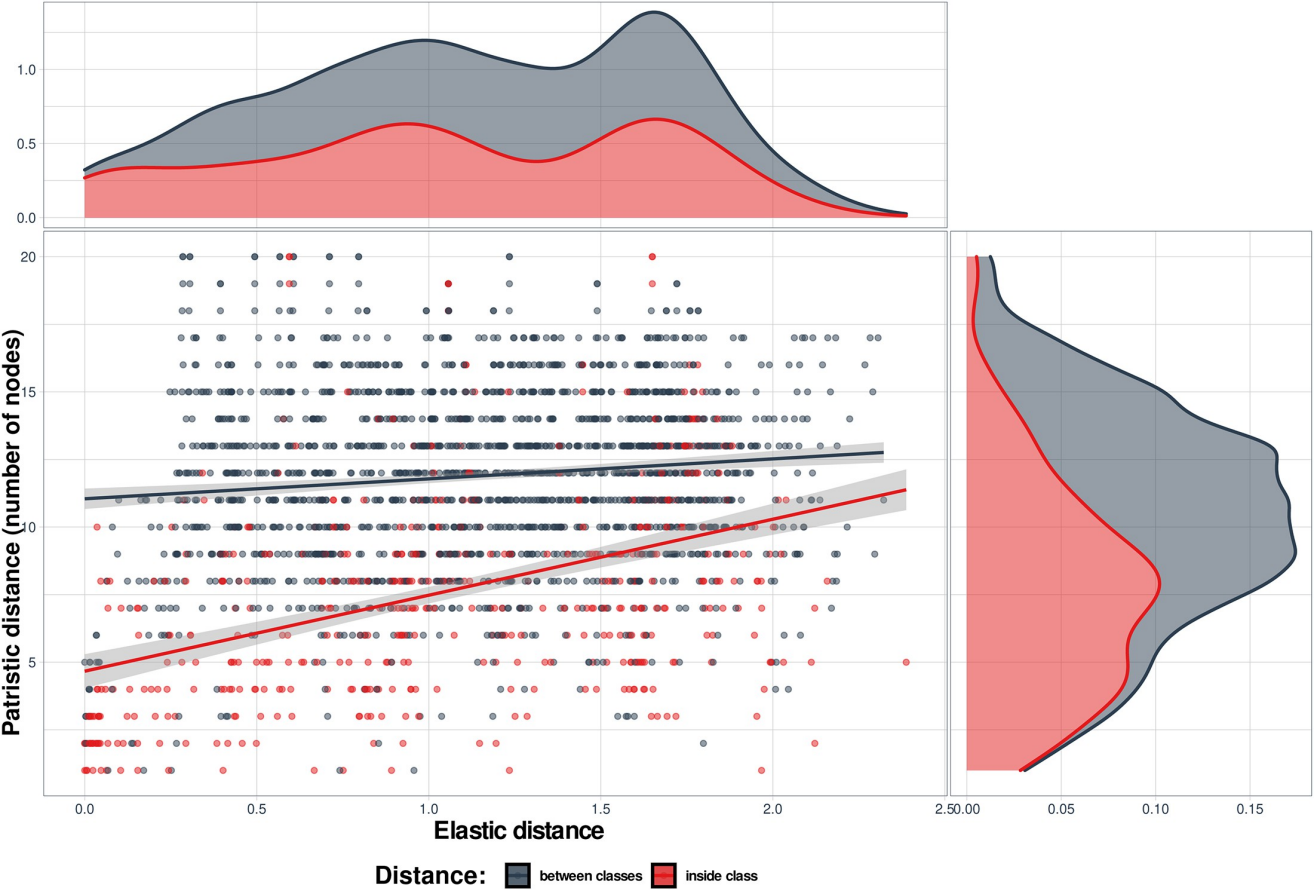
Comparison of elastic and patristic 2 distances between coronavirus genomes. The DNA sequences were grouped according to their taxonomy. Genome pairs where both genomes belong to the same group are highlighted in pink, the inter-group pairs—in gray. Shadows along the regression lines depict 95% confidence limits. The distribution of the values of elastic distances is presented above the main graph and patristic distances on its right side. Within group τ = 0.035, p = 2.2e-16; between groups τ = 0.073, p = 2.2e-16.

**Fig 9:**
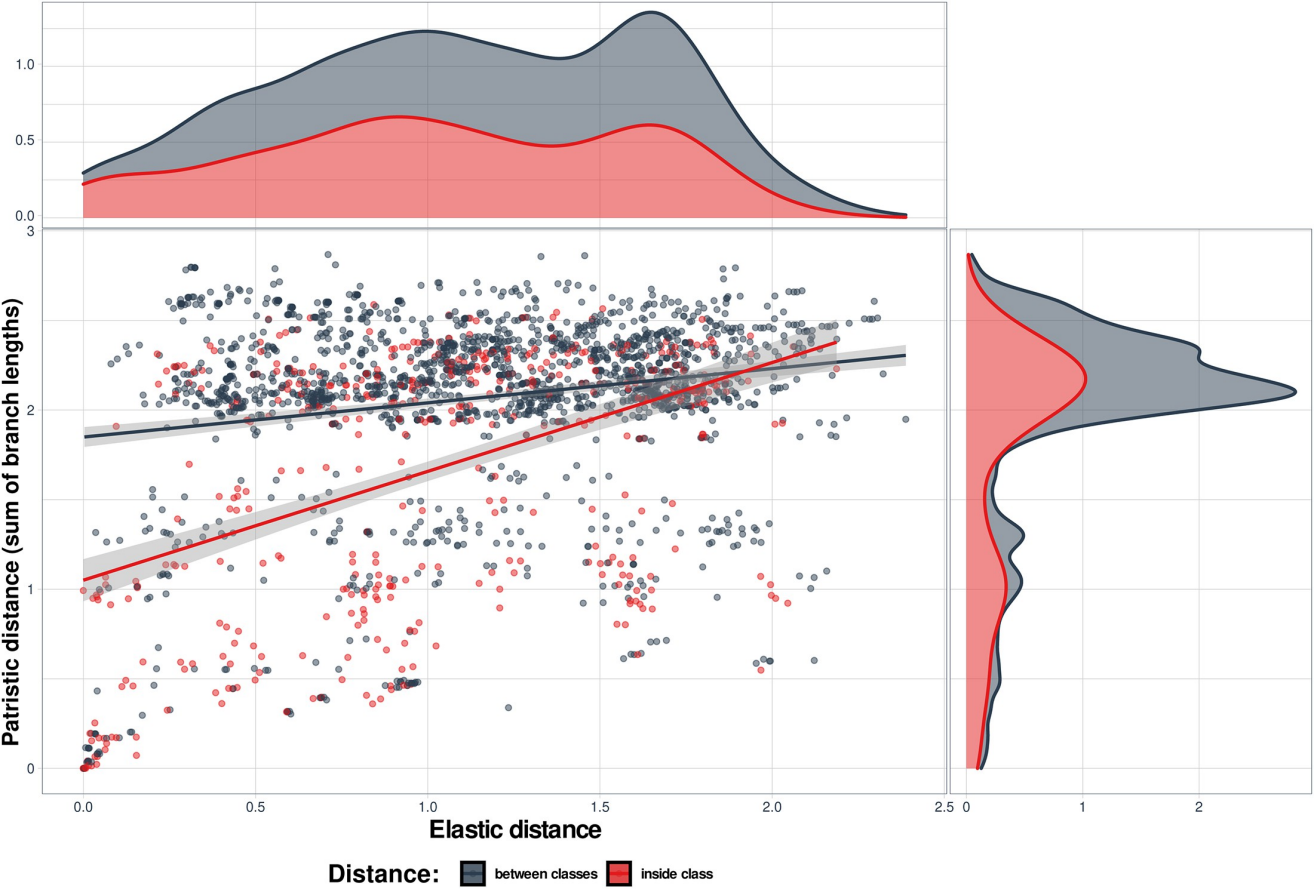
Comparison of elastic and patristic 1 distances between coronavirus genomes. The DNA sequences were grouped according to disease they cause. Genome pairs where the both genomes belong to the same group are highlighted in pink, the inter-group pairs—in gray. Shadows along the regression lines depict 95% confidence limits. The distribution of the values of elastic distances is presented above the main graph and patristic distances on its right side. Within group τ = 0.261, p = 2.2e-16; between groups τ = 0.068, p = 1.47e-05.

Each metric was used to obtain a vector of all possible pair-wise distances through re-dimensioning of a triangular distance matrix sorting it so that all genome pairs have the same order. The comparison involved plotting the genomes in two-dimensional space, where elastic distance was on one coordinate, and one of the patristic distances was on the another coordinate (Figs 8 and 9).

**Fig 10:**
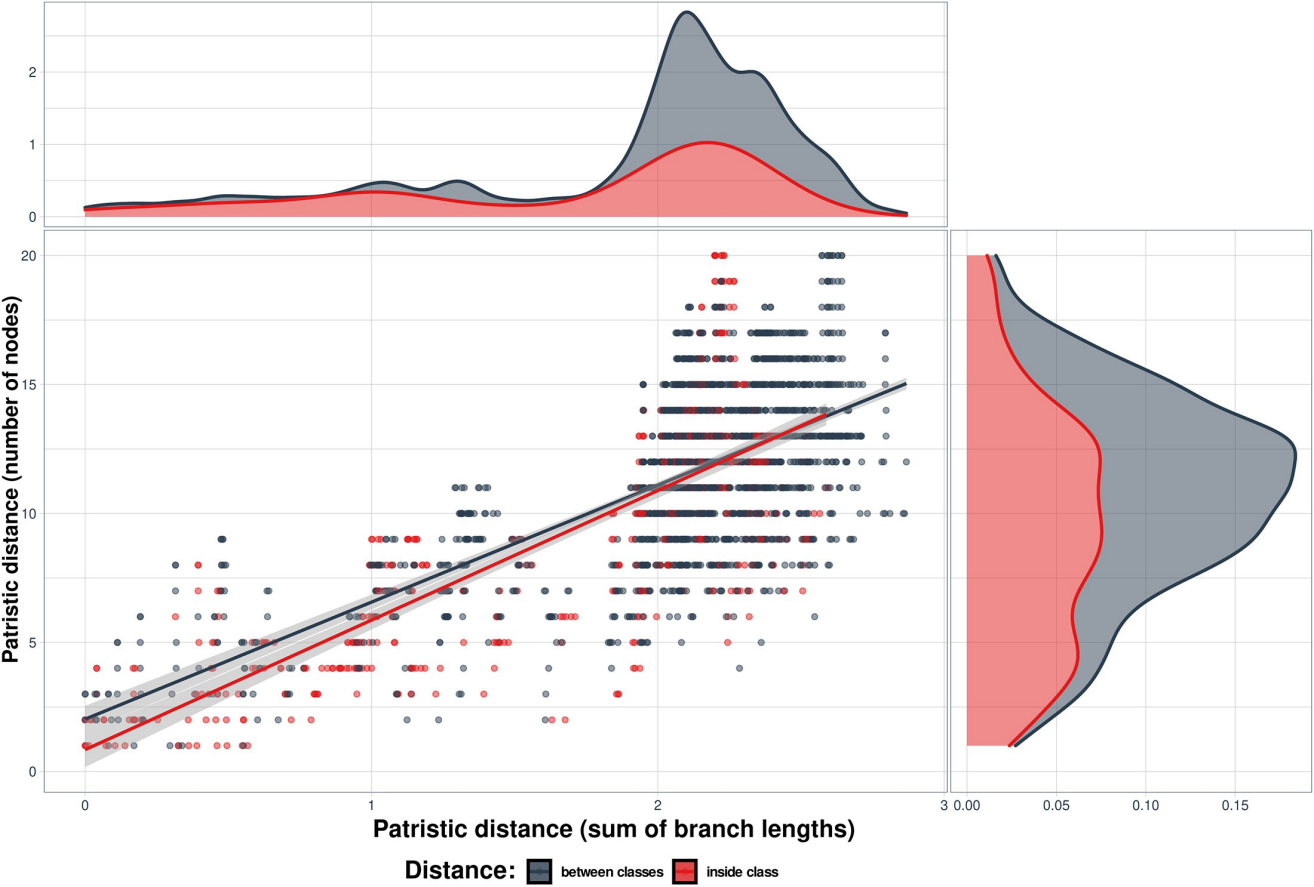
Comparison of patristic distances 1 vs. 2 between coronavirus genomes. The genome sequences were grouped according to disease they cause. Genome OTU pairs where the both genomes belong to the same group are highlighted in pink, the inter-group pairs—in gray. Shadows along the regression lines depict 95% confidence limits. The distribution of the values of elastic distances is presented above the main graph and patristic distances on its right side. Within group τ = 0.567, p = 2.2e-16; between groups τ = 0.410, p = 2.2e-16.

**Fig 11:**
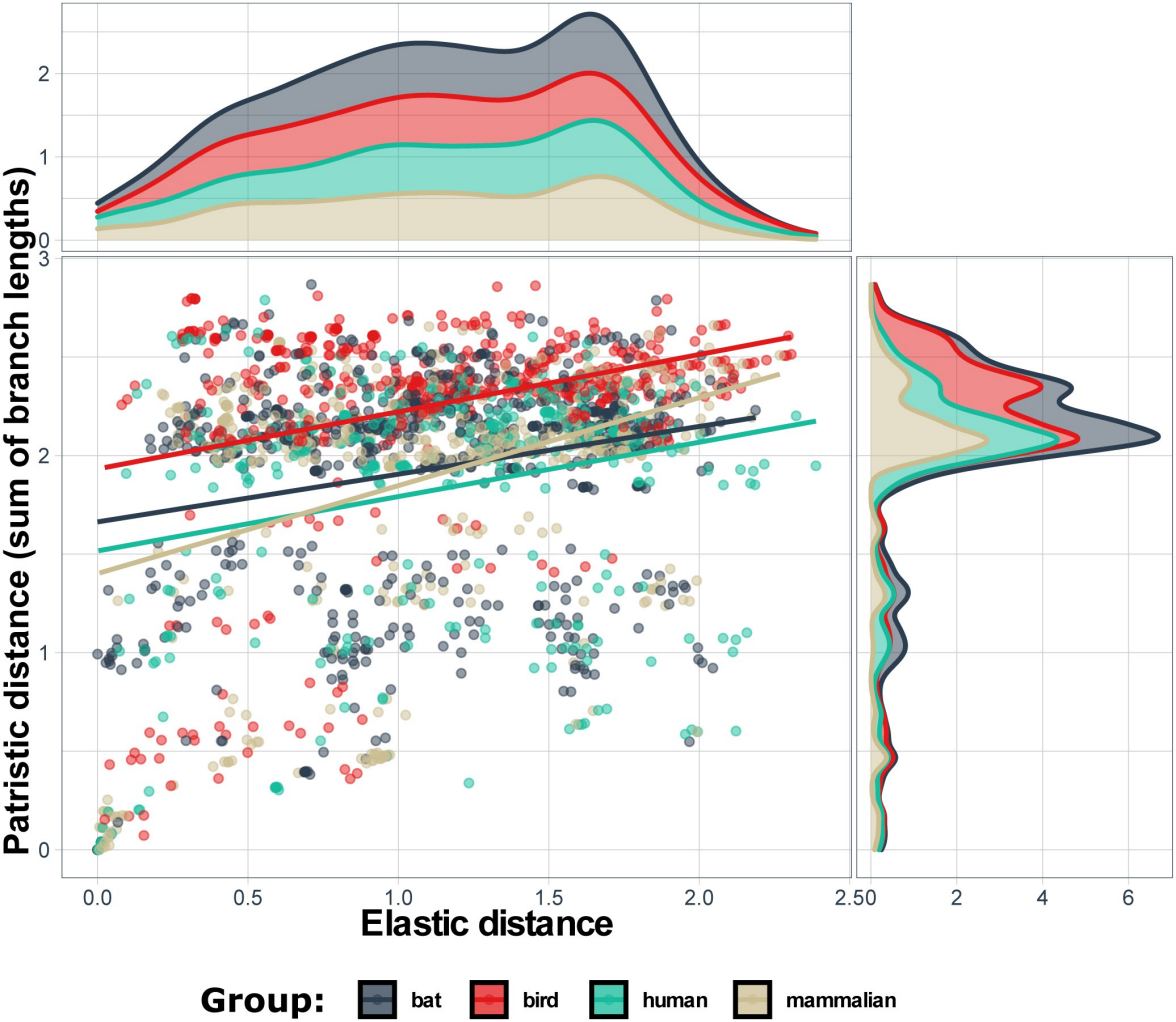
Relationship between pairwise elastic and patristic distances 1 (where the lengths of branches connecting the members of a pair are summed). Only the distances between genomes belonging to the same group according to the disease they cause are presented here. The distribution of the values of elastic distances is presented above the main graph and patristic distances on its right side. Regression lines are colored according to the six disease groups explained in the legend within graph.

It is important to note the difference between the elastic and patristic distances: while they correlated when they were considered only within cluster, there was almost no correlation between them when only intercluster distances were considered (Table 2). As expected, the both regressions were much closer to each other in case of the comparison between the two patristic distances (Fig 10, Table 2). The regression lines are almost parallel to each other if both distances are analyzed within group (Fig 11, Table 2). Therefore, the interesting property of elastic maps is that they bring together specifically the genomes that belong to viruses which changed their important characters. This may emphasize a potential usefulness of using both evolutionary and elastic inferences to predict the possibility of dramatic events in evolutionary trajectories of the viruses.

**Table 2:** Kendall correlation coefficient *τ* for different distance metrics within and between coronavirus genomes.

X	Y	τ	p-value
Patristic1	Elastic	0.117	< 2.2e-16
Patristic2	Elastic	0.123	< 2.2e-16
Patristic1	Patristic2	1.000	0
Taxonomic identity			
Patristic1 intergroup	Elastic intergroup	0.073	2.634e-06
Patristic1 intragroup	Elastic intragroup	0.352	< 2.2e-16
Patristic2 intergroup	Elastic intergroup	0.063	4.224e-05
Patristic2 intragroup	Elastic intragroup	0.326	< 2.2e-16
Group of Disease			
Patristic1 intergroup	Elastic intergroup	0.068	1.465e-05
Patristic1 intragroup	Elastic intragroup	0.261	< 2.2e-16
Patristic2 intergroup	Elastic intergroup	0.069	2.142e-05
Patristic2 intragroup	Elastic intragroup	0.272	< 2.2e-16

In general, while phylogenetic analysis emphasizes the temporal aspect of trait evolution, the elastic mapping may help to reveal other aspects of trait evolution such as the ease of functional transition between trait states etc.

## Discussion

Comparison of sequences representing biological macromolecules is a key tool in genomics, genetics, and bioinformatics. Historically, alignment has become the first and most common approach to compare nucleotide or amino acid sequences with admissible errors. This method has a number of essential disadvantages. The first of them is an arbitrariness in the choice of penalty functions for admissible errors; the second most important disadvantage is the divergence of the methods. There are other disadvantages (for example, high computational complexity), which are well-discussed in [44–46]. Attempts to develop methods that do not use alignment have been undertaken for a long time. The widespread use of such alignment-free methods is hindered by the lack of a reasonable system for comparing results obtained by different methods. This is largely due to the difficulty of selecting an appropriate genetic material.

The genomes of coronaviruses are a very good object for comparative analysis of alignment-based and alignment-free sequence comparison methods. In this study, an attempt was made to compare the internal structuring of 69 genomes of coronaviruses revealed by different comparison methods: 1) traditional alignment with subsequent construction of phylogeny, 2) unsupervised classification based on *K*-means, and 3) the modern method of nonlinear statistics based on elastic maps. The comparison has shown a high efficiency of each of these methods. In general, the clusters identified by the phylogenetic tree based on the multiple sequence alignment were in a good agreement with the classes identified by *K*-means, and a layered graph obtained from a set of classifications with an increase in the number of classes was a structure that is completely natural from a biological point of view. It should be emphasized that the classification using *K*-means and clustering by the method of elastic maps were carried out in the space of frequencies of triplets determined throughout the entire genomes.

The most important difference between the two approaches is that the calculation of the elastic distances does not involve the assumption that they increase with the time while any phylogenetic difference is based on it. Therefore, any homoplasies (multiple independent changes of character state in course separate lineages over the evolution) simply contradict the set of assumptions used. This indeed can impede the application of phylogenies in case of fast state-changing traits, which may be of primary importance. In this study we have used the type of disease caused by a coronavirus as an example of such a trait. Permutation test shows that there is still significant phylogenetic signal in it. On the contrary, some of these genomes being far from each other on the phylogeny still belong to the same cluster on the elastic map, while similar states of the character often appear in different clades, and the same evolutionary clade involves the representatives of different “elastic” clusters (Fig 7). For instance, human HCoVs NL63 (alphacoronavirus) and HKU1 (betacoronavirus), common moorhen HKU21 (deltacoronavirus) and minkWD1127 (alphacoronavirus) formed cluster 5; three avian gammacoronaviruses (IBV, IBVB, and TCoV) and the hedgehog betacoronavirus Erinaceus_VMC formed cluster 6. The genomes of two avian deltacoronaviruses white eye HKU16 and bulbul HKU11, human betacoronavirus SARS-CoV and bat coronavirus BM48_31 formed cluster 2, but this cluster behaved adequately in multidimensional space, in the sense that it was close to the clusters formed by human betacoronaviruses SARS-CoV-2 and MERS-CoV, and was also quite close to the cluster formed by deltacoronaviruses.

Thus, the simultaneous application of the both approaches to the same data set may result in detecting the details which would remain unnoticed otherwise. It is important that the combined approach may enable one to hypothesize that in the case of evolutionary distant genomes but still belonging to that same cluster on elastic map some of them is likely to change the state of this trait soon. In case of the group of diseases, this means, that this virus may be “ready” to change the type of disease caused or/and to change the host. For example, delta coronavirus Night.heron_HKU19, causing a bird respiratory disease (clade 1, Fig 9) had a small interstrain distance on the elastic map with beta coronaviruses Bat_RaTG13, Pangolin_PCoV_GX_P5E, Pangolin_MP789, Beijing.IVDC_03, and Beijing.IVDC_01 (clade 4 that includes also SARS_CoV_2, Fig 9), but a large patristic1 distance (Table 3). Perhaps, more attention should be paid to the Night.heron_HKU19 coronavirus as a virus that can potentially change its host. This is only an example on how data obtained using alignment-free methods can be used and interpreted, and we hope that epidemiologists and virologists can infer more additional information from the results based on the alignment-free method described in this paper.

**Table 3:** Coronavirus strains with a small interstrain distance on the elastic map, but a large patristic1 distance.

OTU1	OTU2	Elastic	Patristic1
Night.heron_HKU19	Bat_RaTG13 0.3121912	0.312	2.797
	Pangolin_PCoV_GX_P5E	0.298	2.782
	Pangolin_MP789	0.314	2.797
	Beijing.IVDC_03	0.324	2.795
	Beijing.IVDC_01	0.324	2.795

The method of elastic maps revealed nonlinear relationships between genomes in addition to linear relationships revealed by *K*-means. The clusters identified by the elastic maps method also coincided well with both the classes of the last layer of the layered graph obtained by *K*-means and clusters revealed by the phylogenetic tree based on the traditional alignment.

In general, phylogenetic relationships found between coronaviruses in our study were in agreement with those based on a set of 68 sarbecoviruses [47] and with phylogenetic network based on 46 betacoronaviruses [48].

## Conclusions

Although, based on a very specific set of genomic data, the obtained results demonstrated that alignment-free comparison methods being free from informal knowledge acquisition have high computational performance and provide an alternative information that potentially can be very important to the phylogenetic analysis. Application of the elastic maps results in clustering DNA sequences that may reflect not only the evolution of clades but also some other hidden features and, thus, providing additional information to the evolutionary analysis based on alignment and molecular evolution model. Noteworthy, alignment-free methods have a significant advantage in computational complexity and do not depend on the quality of alignment. Apparently, the use of alignment-free comparison methods along with molecular phylogenetic analysis can provide additional information about sequences and help to solve various biological problems.

## Supporting information

S1 Table Coronavirus genomes used in the study and their names, NCBI GenBank accession numbers, type of diseases and taxonomy. (XLSX)
